# Breakthrough Cancer Pain Clinical Features and Differential Opioids Response: A Machine Learning Approach in Patients With Cancer From the IOPS-MS Study

**DOI:** 10.1200/PO.20.00158

**Published:** 2020-11-04

**Authors:** Francesco Pantano, Paolo Manca, Grazia Armento, Tea Zeppola, Angelo Onorato, Michele Iuliani, Sonia Simonetti, Bruno Vincenzi, Daniele Santini, Sebastiano Mercadante, Paolo Marchetti, Arturo Cuomo, Augusto Caraceni, Rocco Domenico Mediati, Renato Vellucci, Massimo Mammucari, Silvia Natoli, Marzia Lazzari, Mario Dauri, Claudio Adile, Mario Airoldi, Giuseppe Azzarello, Livio Blasi, Bruno Chiurazzi, Daniela Degiovanni, Flavio Fusco, Vittorio Guardamagna, Simeone Liguori, Loredana Palermo, Sergio Mameli, Francesco Masedu, Teresita Mazzei, Rita Maria Melotti, Valentino Menardo, Danilo Miotti, Stefano Moroso, Gaetano Pascoletti, Stefano De Santis, Remo Orsetti, Alfonso Papa, Sergio Ricci, Elvira Scelzi, Michele Sofia, Federica Aielli, Alessandro Valle, Giuseppe Tonini

**Affiliations:** ^1^ Medical Oncology Department, Campus Bio-Medico University of Rome, Rome, Italy; ^2^ IRCCS Istituto Nazionale Tumori, Milan, Italy; ^3^ Anesthesia and Intensive Care and Pain Relief and Supportive Care, La Maddalena, Palermo, Italy; ^4^ Molecular and Clinical Medicine Medical Oncology, La Sapienza University of Rome, Rome, Italy; ^5^ Anesthesiology, Resuscitation, and Pain Therapy Department, National Cancer Institute, IRCCS Foundation Pascale, Naples, Italy; ^6^ Palliative Care, Pain Therapy, and Rehabilitation, National Cancer Institute, IRCCS Foundation, Milan, Italy; ^7^ Palliative Care and Pain Therapy Unit, Careggi Hospital, Florence, Italy; ^8^ Primary Care Unit, ASL RM1, Rome, Italy; ^9^ Department of Clinical Science and Translational Medicine, University of Rome Tor Vergata, Rome, Italy; ^10^ Second Medical Oncology Division, Città della Salute e della Scienza Hospital of Turin, Turin, Italy; ^11^ Medical Specialties Department, Oncology and Oncologic Hematology, ASL 13 Mirano, Venice, Italy; ^12^ Medical Oncology Unit, ARNAS Ospedale Civico Di Cristina Benfratelli, Palermo, Italy; ^13^ Medical Oncology, AORN Cardarelli, Naples, Italy; ^14^ Palliative Care Unit, ASL AL, Casale Monferrato, Italy; ^15^ Palliative Care Unit, Department of Primary and Community Care, ASL 3 Genovese, Genoa, Italy; ^16^ Palliative Care and Pain Therapy Unit, European Oncology Institute IRCCS, Milan, Italy; ^17^ Palliative Care and Pain Therapy Unit, Papa Giovanni XXIII Hospital, Bergamo, Italy; ^18^ Medical Oncology Unit, National Cancer Research Center “Giovanni Paolo II”, Bari, Italy; ^19^ Pain Therapy Unit, “A. Businco” Hospital, ASL 8, Cagliari, Italy; ^20^ Department of Biotechnological and Applied Clinical Sciences, Section of Clinical Epidemiology and Environmental Medicine, University of L'Aquila, L'Aquila, Italy; ^21^ Section of Clinical Pharmacology and Oncology, Department of Health Sciences, University of Florence, Florence, Italy; ^22^ Department of Medicine and Surgery Sciences, University of Bologna, Bologna, Italy; ^23^ PainTherapy, S. Croce e Carle Hospital, Cuneo, Italy; ^24^ Pain Therapy ICS Maugeri, IRCCS Foundation, Pavia, Italy; ^25^ Medical Oncology, Azienda Sanitaria Universitaria Integrata di Trieste, Trieste, Italy; ^26^ Medical Oncology, Azienda Sanitaria Universitaria Integrata di Udine, Udine, Italy; ^27^ Palliative Care and Oncologic Pain Service, S. Camillo-Forlanini Hospital, Rome, Italy; ^28^ Pain Medicine Unit, S. Camillo-Forlanini Hospital, Rome, Italy; ^29^ Pain Relief, A.O. Dei Colli, Monaldi Hospital, Naples, Italy; ^30^ Division of Medical Oncology, Department of Oncology, S. Chiara University Hospital, Pisa, Italy; ^31^ Medical Oncology, Castelfranco Veneto Hospital, Treviso, Italy; ^32^ Department of Palliative Care, Hospice and Pain Therapy Unit, “G. Salvini” Hospital, Milan, Italy; ^33^ Department of Biotechnological and Applied Clinical Sciences, University of L'Aquila, L'Aquila, Italy; ^34^ Palliative Care, FARO Foundation, Turin, Italy

## Abstract

**PURPOSE:**

A large proportion of patients with cancer suffer from breakthrough cancer pain (BTcP). Several unmet clinical needs concerning BTcP treatment, such as optimal opioid dosages, are being investigated. In this analysis the hypothesis, we explore with an unsupervised learning algorithm whether distinct subtypes of BTcP exist and whether they can provide new insights into clinical practice.

**METHODS:**

Partitioning around a k-medoids algorithm on a large data set of patients with BTcP, previously collected by the Italian Oncologic Pain Survey group, was used to identify possible subgroups of BTcP. Resulting clusters were analyzed in terms of BTcP therapy satisfaction, clinical features, and use of basal pain and rapid-onset opioids. Opioid dosages were converted to a unique scale and the BTcP opioids-to-basal pain opioids ratio was calculated for each patient. We used polynomial logistic regression to catch nonlinear relationships between therapy satisfaction and opioid use.

**RESULTS:**

Our algorithm identified 12 distinct BTcP clusters. Optimal BTcP opioids-to-basal pain opioids ratios differed across the clusters, ranging from 15% to 50%. The majority of clusters were linked to a peculiar association of certain drugs with therapy satisfaction or dissatisfaction. A free online tool was created for new patients’ cluster computation to validate these clusters in future studies and provide handy indications for personalized BTcP therapy.

**CONCLUSION:**

This work proposes a classification for BTcP and identifies subgroups of patients with unique efficacy of different pain medications. This work supports the theory that the optimal dose of BTcP opioids depends on the dose of basal opioids and identifies novel values that are possibly useful for future trials. These results will allow us to target BTcP therapy on the basis of patient characteristics and to define a precision medicine strategy also for supportive care.

## INTRODUCTION

Breakthrough cancer pain (BTcP) is a common event that affects a considerable proportion of patients with cancer.^1^ A variety of definitions for BTcP have been proposed^2,3^. According to the Italian Oncologic Pain Survey (IOPS) study group,^4^ BTcP should be defined “as a relevant change in pain intensity of severe intensity in patients who receive an effective treatment with opioids.”^4^^(p963)^ Nevertheless, despite this unique definition, BTcP encloses a wide range of manifestations that differ, among other features, in intensity, duration, frequency, and triggering events. BTcP represents a clinically relevant condition with a negative impact on the patient’s quality of life. In the majority, it is difficult to achieve an acceptable degree of relief because patients with cancer have complex pain syndromes. These patients often require more intense therapeutic protocols and, therefore, more time may be required to achieve adequate pain control.^5^

At present, several gaps exist in the knowledge of BTcP management. These partially unanswered questions, among others, concern the optimal drug administration route, pharmacokinetics, the balance between rapid-onset and slow-onset opioids, and the eventual difference of BTcP response deriving from clinical features, such as stage, primary site, or metastases.

In this analysis, it was thus hypothesized that the unique BTcP definition might actually enclose diverse pathologic entities, possibly with peculiar clinical needs and specific responses to drugs. To explore this supposition, we used novel multiparametric artificial intelligence algorithms that can simultaneously analyze different clinical features and identify the existence of shared patterns. These so-called unsupervised learning algorithms have already been extensively used, for example, for the identification of breast cancer subtypes.^6^ Nevertheless, to our knowledge, no authors have yet tried to explore the issue of BTcP management using these techniques.

To fulfill this purpose, we used data that were collected by the IOPS group in a large, multicentric national study^5,7,8^ that enrolled 4,056 patients from 32 centers with opioid-controlled basal pain suffering from BTcP. This work is therefore a secondary analysis of the IOPS group survey that aims to identify novel subtypes of BTcP through the use of unsupervised learning algorithms.

## METHODS

### Patients’ Enrollment and Data Collection

Details concerning the enrollment of patients are extensively described in the main paper from the IOPS group.^5^ In brief, local ethical committees approved the protocol, and written informed consent was obtained from each patient. Interviews were performed in different settings, in particular, oncology, pain therapy, palliative care, and radiotherapy. Patients were ≥ 18 years of age; diagnosed with cancer at any stage; had stable background pain in the last week with an intensity of, at most, 4 on a numerical scale from 0 to 10; and had episodes of BTcP with an intensity ≥ 5, clearly distinguished from background pain. The definition of BTP was a transitory pain exacerbation of moderate to severe intensity that occurs spontaneously or is predictably well distinguished from background pain. Exclusion criteria were the absence of a cancer diagnosis, uncontrolled background pain (> 4 on a numerical scale of 0 to 10), or no relevant increases in pain intensity (< 5) that could be interpreted as BTcP episodes. Patients who were unable to provide information about the data required for the study as a result of either cognitive failure or terminal disease were also excluded. A comprehensive list of clinical variables was collected for each patient that consisted of basal pain and BTcP site, duration, frequency, intensity, relieving factors, triggers, drugs, primary cancer site and stage, and concomitant symptoms for a total of 1,086 variables. Interviews were registered by collecting personnel in a closed online form and centrally stored.

### Therapy Satisfaction

The association of each clinical feature with satisfaction toward BTcP therapy was investigated using a simple logistic regression. Therapy satisfaction was expressed as a binomial outcome. We used the false discovery rate method^9^ to correct *P* values for multiple comparisons. Features with less than 5% of missing data and associated with a corrected *P* value of < .1 and, for categorical features, a log_2_ odds ratio greater than 1 were used to build a multivariable logistic regression. To investigate the correlation—simultaneously for all patients and on the same scale—between the amount of opioids used and BTcP therapy satisfaction, all doses of opioid drugs were converted^10^ to equivalent intravenous morphine doses and expressed as a total daily dose: one for BTcP-directed opioids and for basal pain opioids. Doses were converted to intravenous morphine doses and not to oral morphine doses because intravenous morphine has been increasingly used in different clinical settings and would provide more interpretable graphics in Results. Moreover, to explore the interaction of fast-acting and long-acting opioid dosages, we calculated for each patient the BTcP opioids–to–basal pain opioids ratio (OpR). A polynomial logistic regression was used to catch nonlinear relationships between opioid doses and therapy satisfaction.

### Cluster Computation and Visualization

Features defining the clinical characteristics of BTcP were selected to perform clusters computation. Features with missing data that accounted for more than 5% of patients were excluded. The above-mentioned features were used to calculate a dissimilarity matrix using a cluster package^11^; as features were also composed of non-numeric variables, we used the Gower metric^12^ for dissimilarity matrix calculation. Partitioning around the k-medoids^13^ algorithm was used to compute clusters using the dissimilarity matrix as an input. The algorithm was run different times using a range of cluster numbers (2-30). Silhouette statistics^14^ were calculated for each run, and the optimal number of clusters was manually picked as being that with the best trade-off between silhouette statistics and reasonable clinical interpretation. We used complete-linkage hierarchical clustering with 12 clusters, along with Rand index calculation, to explore the replicability of clusters with a different algorithm.

*t*-Distributed stochastic neighbor embedding^15^ algorithm was used to project dissimilarities between patients in a bidimensional space, with closer points representing patients with more similar clinical BTcP features. An online tool allows for the repetition of the performed classification on new sets of patients.^16^

### Clusters Analysis

*t*-Test, Mann-Whitney test, and χ^2^ test were used to assess the association of parametric, nonparametric, and categorical features, respectively, with each cluster. The false discovery rate method was used to correct *P* values for multiple comparisons. Therapy satisfaction was investigated separately for each cluster, as previously described, for all samples.

### Data Handling

Data were imported and analyzed using R (v3.5.2).^17^ The following packages were used for the analyses: dplyr,^18^ cluster,^11^ Rtsne,^19^ ggplot2,^20^ gmodels,^21^ Rmisc,^22^ epiR,^23^ mgcv,^24^ knitr,^25^ RColorBrewer,^26^ and mgcViz.^27^

## RESULTS

### Patient Characteristics

A total of 4,016 patients were enrolled in the study during a period of 24 months. Men accounted for 54.8% of the total, and mean age was 64.6 years (range, 18-97 years). The majority of visits were performed for oncologic (52.0%) and pain therapy (29.5%) purposes. Together, inpatient (37.3%) and outpatient (34.3%) settings accounted for more than one half of visits. The most common cancer primary organs were the lung (24.0%), breast (11.3%), pancreas (8.3%), and colon 7.5%. Mean Karnofsky performance status was 48. According to the inclusion criteria, basal pain was generally controlled; mean basal pain numeric rating scale was 3.0. Patients’ characteristics are listed in Table 1.

**TABLE 1.**
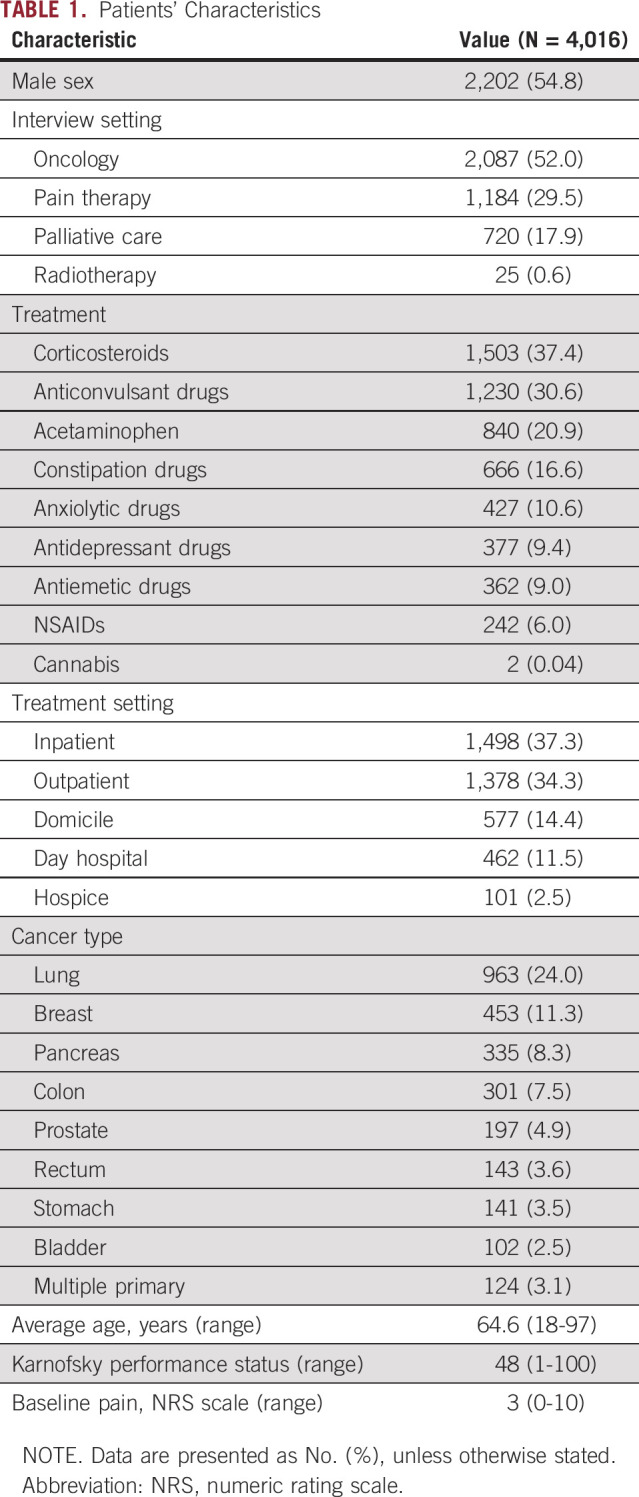
Patients’ Characteristics

### Cluster Computation

**FIG A1.**
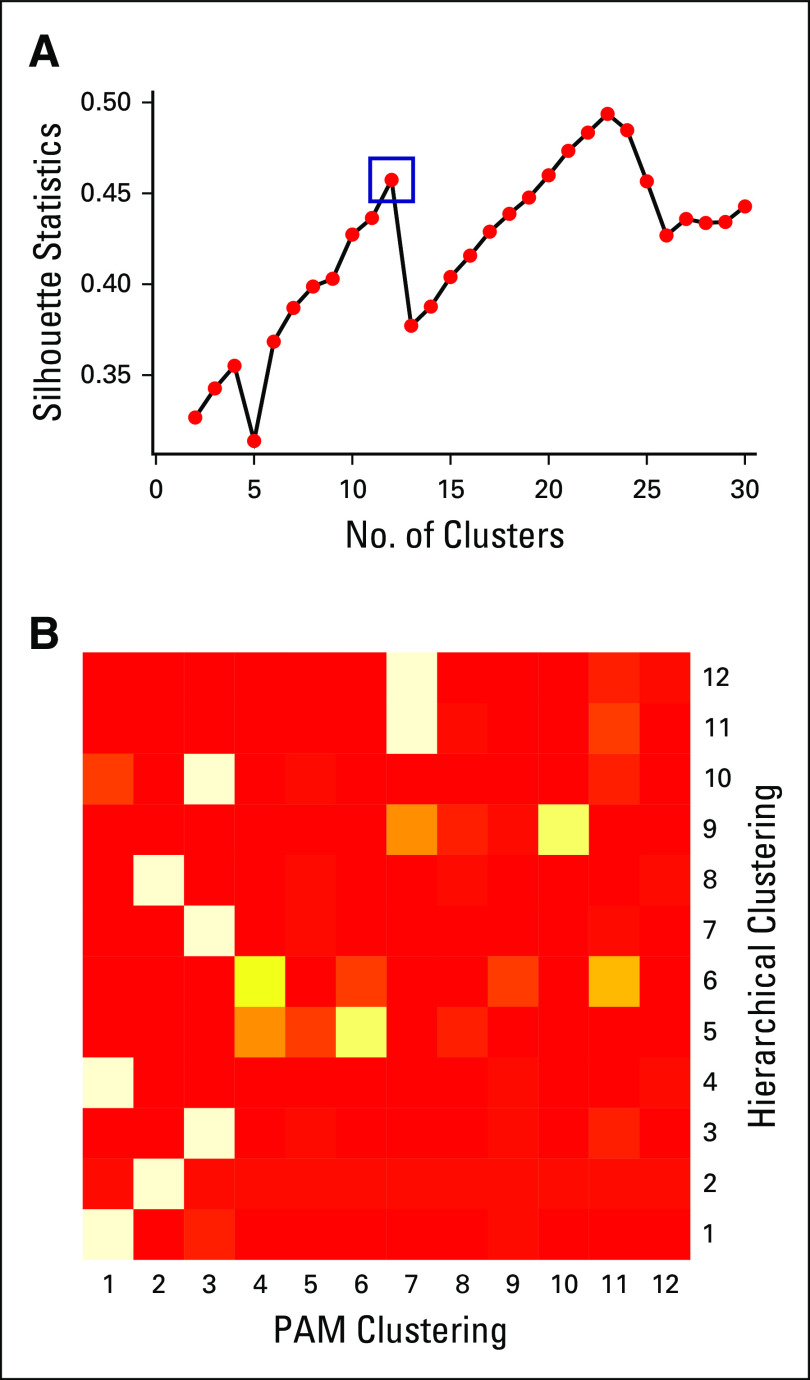
(A) Silhouette statistics for clusters 2 to 30. The appropriate number of clusters for additional analyses was found to be 12, an optimal trade-off between the average width of clusters silhouette (0.45) and the interpretability of clusters themselves. (B) Heatmap showing the concordance of PAM clustering with complete-linkage hierarchical clustering. The Rand index, which compares the replicability of the two algorithms, was 0.89.

To investigate whether subtypes of BTcP exist, we used BTcP features to build an unsupervised clustering model. The number of BTcP episodes, BTcP peaks duration, BTcP type, BTcP intensity, number of days since the beginning of BTcP episodes, the eventual benefit from pharmacotherapy, the eventual benefit from rest, and whether BTcP was enhanced by movements were the eight BTcP-defining variables selected for the final model, which was built with the k-medoids algorithm. We chose 12 as an optimal trade-off between the average width of clusters silhouette (0.45; Appendix Fig A1A) and the interpretability of the clusters themselves. The average internal dissimilarity was acceptably low, ranging between 0.05 and 0.16. A Rand index of 0.89 was obtained comparing results from PAM (partition around medoids) clustering and hierarchical clustering, which suggests good stability of the partitioning with different methods (Appendix Fig A1B).

**FIG 1.**
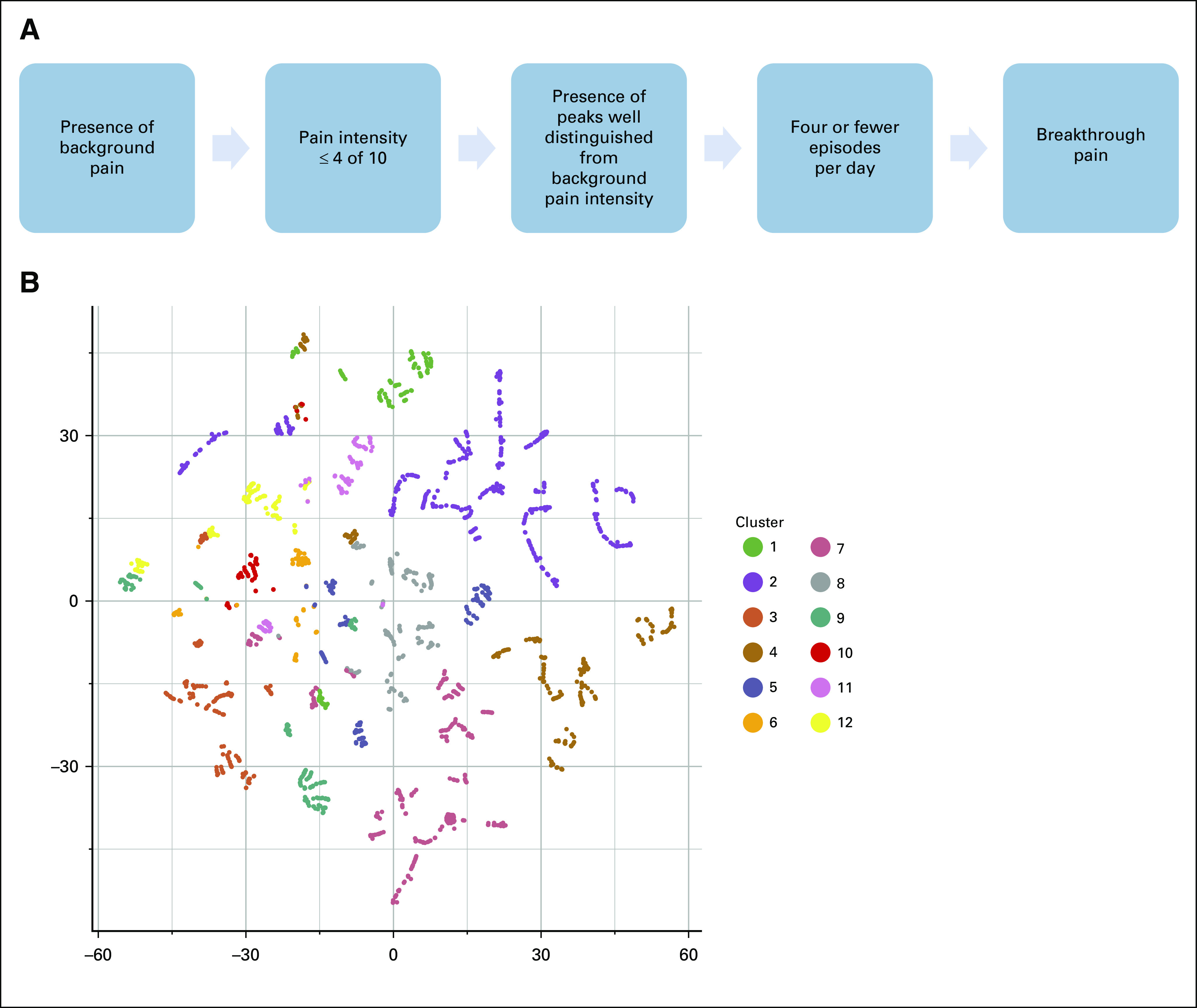
(A) Algorithm used for the diagnosis of breakthrough cancer pain (BTcP) during patients’ enrollment in the Italian Oncologic Pain Survey (modified from Mercadante et al^8^). (B) A two-dimensional *t*-distributed stochastic neighbor embedding projection of all patients, colored by their clusters, on the basis of the following BTcP features: number of BTcP episodes, BTcP peaks duration, BTcP type, BTcP intensity, number of days since the beginning of BTcP episodes, eventual benefit from pharmacotherapy, eventual benefit from rest, and whether BTcP was enhanced by movements. Each point represents a patient. Patients’ dissimilarity in BTcP clinical features is represented by the points distance. Colors represent 12 clusters computed through partitioning around the medoids (k-medoids) algorithm.

Figure 1 shows the algorithm used to define BTP (Fig 1A) and a 2-dimensional *t*-distributed stochastic neighbor embedding projection of all patients, colored by their clusters (Fig 1B). An online tool is available for the classification of new patients according to our method.^16^

### Characteristics of BTcP Clusters

**FIG 2.**
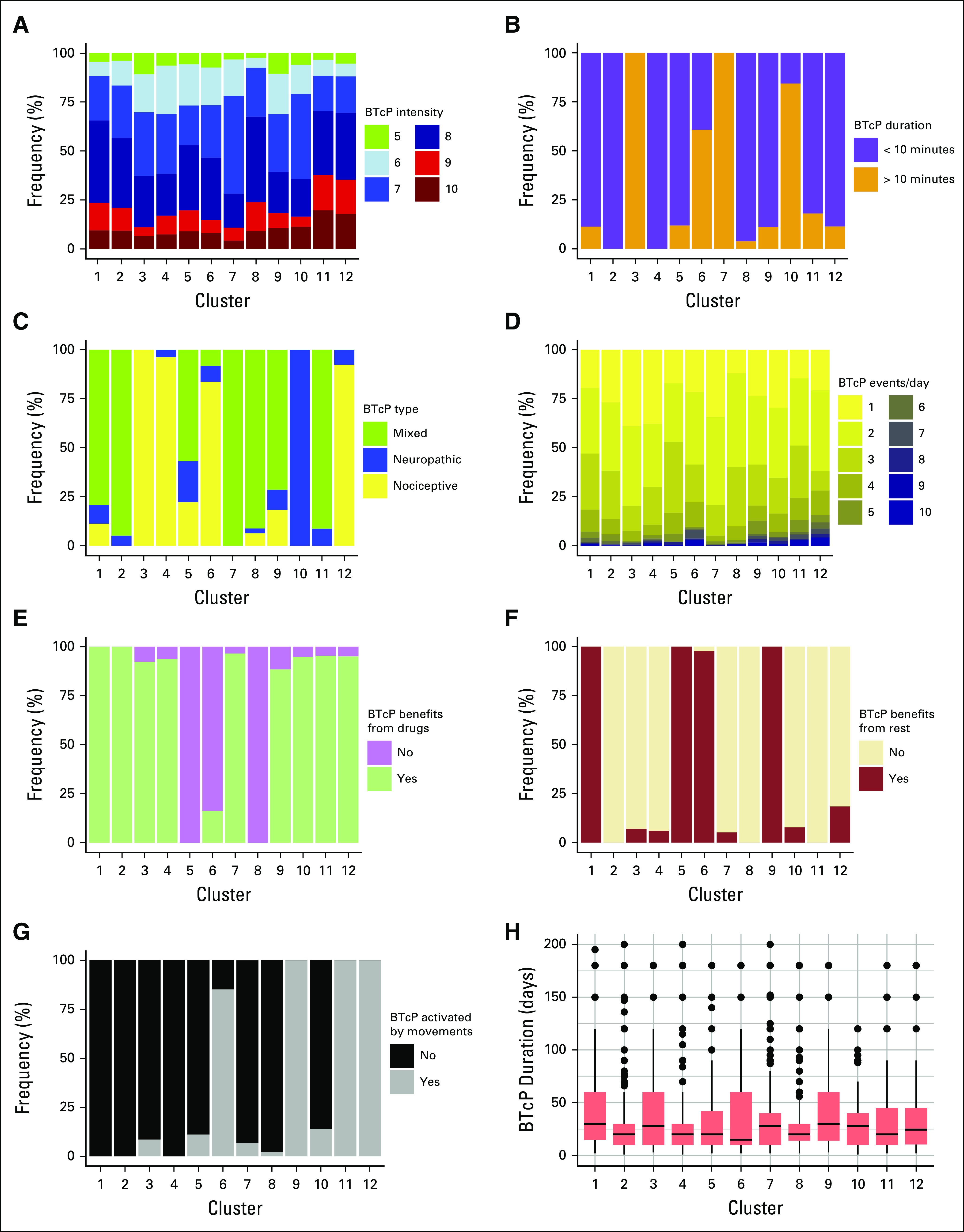
Defining features of the 12 breakthrough cancer pain (BTcP) clusters. Plots represent in order: (A) BTcP intensity using numeric rating scale, (B) BTcP peak duration, (C) BTcP type, (D) number of BTcP events per day, (E) presence of benefit in BTcP management with pharmacotherapy, (F) presence of benefit in BTcP management with rest, (G) presence of BTcP activation with movements, and (H) days since BTcP episodes started.

We analyzed the enrichment of the eight BTcP-defining variables and of other clinical features among the clusters. A description of each cluster is available in Table 2. A summary of BTcP features according to cluster is presented in Figure 2.

**TABLE 2.**
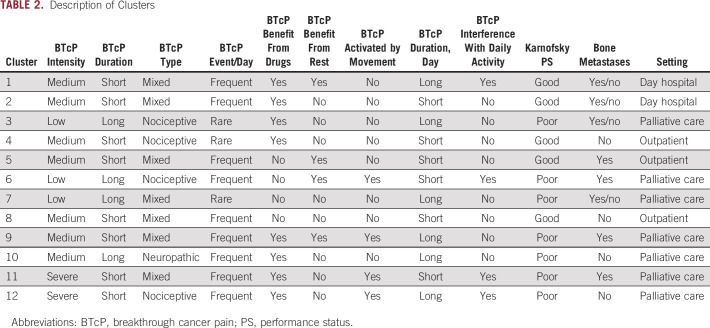
Description of Clusters

### BTcP Therapy Satisfaction

**FIG 3.**
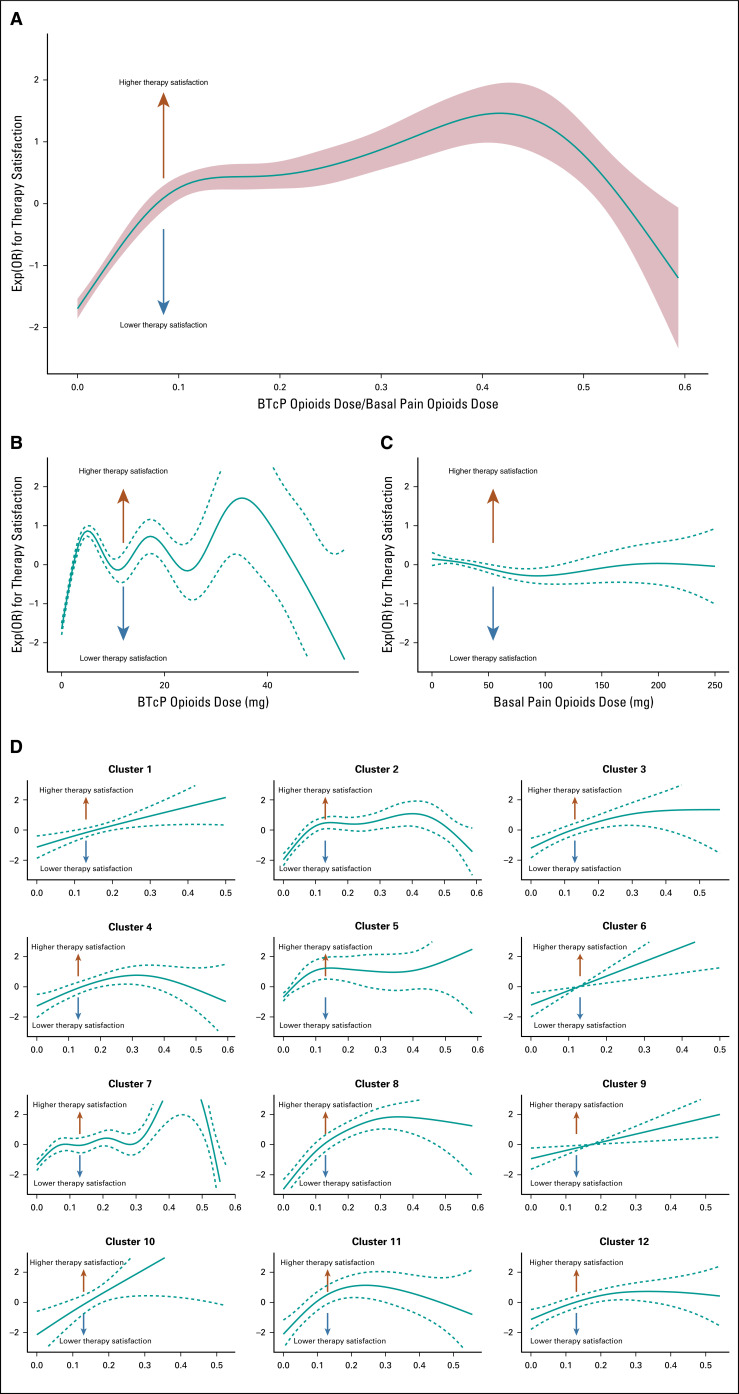
(A) Correlation of breakthrough cancer pain (BTcP) therapy satisfaction with BTcP opioid dose and basal pain opioid dose ratio. (B) BTcP opioid drug dose alone, and (C) basal pain opioid drug dose. Solid lines represent logistic regressions calculated with more than 1 degree of freedom and dashed lines represent 95% CIs. (D) Correlation between fast to basal opioids ratio and therapy satisfaction for each cluster. Exp(OR), exponent (odds ratio).

Finally, we tried to assess what influenced patient-reported BTcP therapy satisfaction. After converting the opioid dose to a unique scale—corresponding to the equivalent dose of intravenous morphine—we investigated using a nonlinear model the influence of basal opioid dose, BTcP opioid dose, and OpR on patient-reported therapy satisfaction. Of interest, whereas the basal opioid dose did not demonstrate any great impact on therapy satisfaction (Fig 3C), and BTcP opioid dose showed some irregular peaks of satisfaction with CIs that often reached the indifference line (Fig 3B), OpR seemed to depict a clear, optimal peak between 0.4 and 0.45 (Fig 3A). This roughly corresponds to a daily dose of sublingual fentanyl 100 μg for BTcP and a daily dose of oral morphine 30 mg as the basal opioid dose.

We separately performed the same analyses on previously defined clusters (Fig 3D). Of interest, not all the clusters showed the same relationship between OpR and satisfaction. For clusters 1, 6, and 10, the satisfaction seemed to grow indefinitely with an increase of the OpR opioids, whereas clusters 7, 8, and 11 seemed to have clear, optimal peaks of OpR. Despite the interpretation being challenged by some large CIs, we can say from these data that, depending on the cluster, optimal OpR ranges from 15% to 50%.

## DISCUSSION

This paper demonstrates a novel approach for the investigation of BTcP. Our findings identified 12 subtypes of BTcP with peculiar response to drugs and clinical presentation. We acknowledge that this study was not designed to perform this analysis and, moreover, that the large number of clusters might interfere with their interpretability and clinical utility. Nevertheless, this study represents a proof-of-concept for this investigational approach.

Some of our findings might already provide some indication for future clinical practice. First, it seems that an optimal ratio between BTcP opioids and basal pain opioids exists. Using our same data, another group proposed 0.20 (one fifth) as the optimal ratio.^5^ Nevertheless, they used a frequentistic approach, as 0.20 is simply the most common ratio among the cohort. Our group instead modeled the ratio toward an outcome (BTcP therapy satisfaction) and highlighted a peak of satisfaction within a ratio range of 0.40-0.45. What seems clear, though, is that such an optimal level exists. This possibly suggests that, instead of starting BTcP opioid titration with the lowest possible dosage, as proposed previously,^28^ titration could start immediately with an optimal opioid dosage. Moreover, cluster analysis reveals that this ratio might not be the same for all patients: Some patients might benefit from a higher BTcP opioid dosage (cluster 2 and 7), whereas others might benefit from a lower one (cluster 11). Finally, for some patients, we did not observe an upper threshold for this ratio (cluster 1, 6, and 9), perhaps pointing out patients for whom a strong BTcP opioid dosage increase is required.

The interpretation is made difficult by the multiple associations and, at this stage, is not mature enough to suggest any immediate change in clinical practice. However, we made available an online free tool^16^ that allows for the classification of new patients according to our algorithm and returns a proposed BTcP therapy which depends on the patient cluster optimal OpR and basal opioid dose. We suggest that this tool might be used in the future to prospectively validate the clinical importance of our clusters in clinical practice and to compare our proposed opioid dosages in settings different from ours.

The presence of distinct BTcP phenotypes, each one associated with specific clinical features, could also be a reflection of diverse underlying pathophysiologic mechanisms: Our work suggests that preclinical research might gain insight into these possible differences and help the development of a tailored therapy also for BTcP.

The main limitation of our study is the appropriateness of collected data for the scope of our work. We believe that a prospective study specifically designed for the investigation of BTcP clusters—possibly with long-term follow-up and therapy success outcomes and not limited to a single timepoint evaluation—might enable a clearer identification of distinct clusters. Moreover, our approach lacks an external validation of cluster consistencies and reproducibility. Nevertheless, these limitations do not interfere with the main scope of our paper, which was to offer a proof of concept for an innovative approach for BTcP management.

In conclusion, this work identifies criteria for optimal BTcP opioids therapy personalization and offers a reproducible classification for the enrollment and stratification of patients in future BTcP trials.
